# Cognitive emotion regulation strategies and psychological distress during lockdown due to COVID‐19

**DOI:** 10.1002/ijop.12818

**Published:** 2021-11-07

**Authors:** Jose A. Rodas, Maria F. Jara‐Rizzo, Ciara M. Greene, Rodrigo Moreta‐Herrera, Daniel Oleas

**Affiliations:** ^1^ Universidad de Guayaquil, Faculty of Psychological Sciences University of Guayaquil, Cdla. Universitaria Universidad de Guayaquil Guayaquil Ecuador; ^2^ School of Psychology University College Dublin Dublin Ireland; ^3^ Pontificia Universidad Católica del Ecuador Ambato Ecuador; ^4^ Faculty of Psychology Universidad Autónoma de Madrid Madrid Spain; ^5^ Faculty of Marketing and Comunication Universidad Ecotec Samborondón Ecuador

**Keywords:** Emotion regulation, COVID‐19, Lockdown, Depression, Anxiety

## Abstract

Several governments have implemented strict measures to reduce the spread of COVID‐19, such as lockdown measures. However, these measures have brought negative consequences at an individual level by exacerbating the psychological distress caused by the pandemic. We evaluated the role of cognitive emotion regulation strategies (CERS) on the levels of anxiety and depression during the lockdown in a sample of 663 Spanish‐speaking adults, while controlling for variables related to social support, hobbies, seeking information related to COVID‐19, perceived risk of infection, time of assessment, number of deaths and contagions during the assessment and age. Using multiple regression analyses with a stepwise model selection procedure, 29% of the variance in anxiety and 38% of the variance of depression were found to be predicted by specific CERS. The impact of CERS on anxiety and depression was moderated by the sex of participants and the time of assessment, indicating that CERS did not have the same protective or harmful effects in all participants and situations. Based on our results, recommendations are provided for improving coping with stressful events where lockdown measures are taken.

During 2020 the world faced a pandemic of rapid spread, forcing many governments to implement strict measures to control it. Arguably, the most impactful measure taken by governments to face COVID‐19 has been social distancing, since this measure limits the ability to work and severely reduces the opportunities for social interaction. For example, during the first months of the pandemic, several countries implemented aggressive lockdown restrictions which lasted for several months. These restrictions typically involved the cessation of public transportation, restriction of travel to essential journeys such as those to supermarkets, hospitals and pharmacies and the closure of most work spaces. These measures represent an important challenge for many individuals (Zajenkowski et al., 2020) since the isolation resulting from the lockdown severely hinders access to the social support needed to face the emotional consequences of the pandemic, placing greater emphasis on the individual regulation of emotions.

Inadequate regulation of emotions has been related to the presence of mood and anxiety disorders (Picó‐Pérez et al., 2017), poor mental health (Hu et al., 2014) and reduced well‐being (Kraiss et al., 2020). This highlights the relevance that emotion regulation can have not only for specific mental health issues but also in wellness in general as it affects how a person approaches emotionally impactful events.

Garnefski and Kraaij (2007) have identified nine cognitive strategies that can be used to regulate emotions (see Table 1). Several of these strategies have been related to the presence of negative emotions, such as depression, anxiety, anger and stress (Garnefski & Kraaij, 2006, 2018). Models consisting solely of these strategies can predict a high proportion of variance in emotional state (e.g., 43%; Garnefski & Kraaij, 2006). However, variables specifically related to lockdown measures may also play an important role in the presence of depression and anxiety. The COVID‐19 pandemic represents an unprecedented situation, and the effects of extended lockdowns and social isolation on individuals' emotional response are still unclear. In a study investigating variables that predicted compliance with protective measures to prevent the spread of COVID‐19, Rubaltelli et al. (2020) found that variables such as the number of media sources used to look for information about COVID‐19 (media exposure), perceived risk of becoming infected, and level of education significantly predicted the experience of anxiety.

**TABLE 1:** Cognitive emotion regulation strategies (Garnefski & Kraaij, 2007)

Strategy	Description
Other‐blame	Attributing the responsibility for the event triggering the negative emotions to others
Self‐blame	Attributing the responsibility for the event triggering the negative emotions to oneself
Positive reappraisal	Re‐evaluating the event in a more positive way
Putting into perspective	Putting the event into a broader perspective in order to minimise its relevance
Catastrophising	Focusing on the negative aspects of the situation
Rumination	Continuously bringing the event back to memory
Positive refocusing	Refocusing attention on other more positive things
Refocus on planning	Refocusing attention on possible solutions
Acceptance	Accepting the event and resigning oneself to it

The relationship between social media exposure and the presence of psychological distress during the COVID‐19 outbreak has also been observed in other studies. For example, Gao et al. (2020) observed a significant positive association between perceived exposure to social media and mental health problems, especially anxiety and depression. It is possible that the associations found by Rubaltelli et al. (2020) and Gao et al. (2020) between exposure to media and the presence of psychological distress are due to the negative tone of media coverage during the first months of the pandemic (Aslam et al., 2020) and the daily reporting of new cases and increasing death rates.

One other factor that may affect the psychological impact of lockdown is the availability of social support. Several studies (Baqutayan, 2011; Çivitci, 2015) have observed that participants with better social support can cope more successfully with stressful events. Furthermore, in a study with chimpanzees (Wittig et al., 2016), it was found that social support, provided by interaction with bond partners, reduced stress hormone levels. It is possible that the number of persons in the house during lockdown and having a partner may mediate the negative psychological effects of lockdown since they represent opportunities for social interaction and support during a time where contact with others was restricted. However, it is also possible that having more persons in the same house increases the occurrence of interpersonal conflicts, raising the levels of psychological distress.

In a study conducted in Spain during the first weeks of lockdown (Fullana et al., 2020), several factors were studied as possible behaviours that would help to cope with anxiety and depression symptoms. They found that a healthy diet, following a routine, not reading news about COVID‐19, pursuing hobbies and contact with outdoors were good predictors of lower levels of depression. These results seem to highlight the relevance of daily activities in preventing psychological distress, including having hobbies.

For the present study, cognitive emotion regulation strategies (CERS), behavioural and demographic variables and COVID‐19 cases and death rates were analysed as possible predictors of psychological distress (anxiety and depression symptoms) during the first months of lockdown due to COVID‐19.

## METHODS

The present study has been pre‐registered at https://doi.org/10.17605/OSF.IO/XBGQN on 26 March 2020.

### Participants

The study was completed by 663 Spanish‐speaking participants (417 female, 239 male, 7 preferred not to say; mean age = 30 years [18–75], *SD* = 11.3). Data collection took place during the implementation of severe lockdown measures in Ecuador, between March 26 and 1 June 2020. Most of the sample consisted of Ecuadorian participants (93%). The rest of the sample included participants from other American countries and Europe. No compensation was offered for participation. More detailed information about the sample can be found in Rodas et al. (2021). According to two power analyses conducted with G*Power (Faul et al., 2009), the number of participants used for the multiple regression models (for anxiety and depression) provided 100% power in both models according to the effect sizes found in our analyses. In order to evaluate the impact of time, a categorical variable was created dividing the sample into two groups: Group 1 included participants who responded the questionnaire during the first 15 days of the assessment phase (*n* = 344, mean age = 29.86 years, *SD* = 12.11, min and max age = 18–75 years) and Group 2 included participants who responded during the second round of data collection, between day 42 and 57 (*n* = 313, mean age = 30.11 years, *SD* = 10.33, min and max age = 18–71 years). The remaining six participants were not assigned any code (i.e., missing data).

All procedures performed in the current study were in accordance with the ethical standards of the Comité de Ética de Psicólogos Clínicos de Tungurahua (Ethic's Committee from the Association of Clinical Psychologists from Tungurahua) and with the 1964 Helsinki Declaration and its later amendments or comparable ethical standards. Informed consent was obtained from all individual adult participants included in the study.

### Materials

Participants completed a single questionnaire including four different instruments, in the following order:

The first instrument consisted of a questionnaire covering sociodemographic information and information related to hobbies, medical and psychiatric history, house characteristics, media sources of information accesses, number of times they looked for information about COVID‐19 and concerns during the first few months of isolation due to COVID‐19. This information is not reported in this article. However, all data can be accessed online (https://osf.io/9cr3q/). The number of hobbies was obtained from counting the hobbies participants reported having during lockdown, and perceived risk of infection was obtained using a Likert scale (5 points). An example of the complete form participants were asked to complete can be found at https://osf.io/p9afw/.

The presence of anxiety was assessed with the State–Trait Anxiety Inventory (STAI; Spielberger, 2010). This instrument evaluates the current experience of anxiety (state anxiety sub‐scale) and the presence of anxiety as a personality trait (trait anxiety sub‐scale). Each subscale consists of 20 items, and the participant has to choose between four possible options (scored from 0 to 3) for each item. Higher scores represent higher levels of anxiety. The Spanish adaptation (Buela‐Casal et al., 2015) has shown good criterion validity and good internal consistency, with α scores ranging between .89 and .95. For the present study, only the state anxiety sub‐scale was used.

Participants were also assessed on their use of CERS with the 27‐item Spanish version of the Cognitive Emotion Regulation Questionnaire (CERQ; Garnefski & Kraaij, 2007; Holgado‐Tello et al., 2018). This questionnaire evaluates the use of the nine cognitive emotion regulation strategies proposed by Garnefski and Kraaij (2007). Response options are presented on a 5‐point Likert scale and scores are calculated for each sub‐scale. Higher scores represent greater use of a particular strategy. The Spanish adaptation of this instrument has shown the same 9‐factor structure as the original version, and internal consistency scores ranging between 0.72 and 0.88.

The presence of depression symptoms was assessed with the Center for Epidemiological Studies Depression scale (CES‐D; González‐Forteza et al., 2008). This scale consists of 20 items covering several depression symptoms. Participants are required to rate each item from 0 to 3 using the week prior to the evaluation as a time frame. A total score is obtained and higher scores reflect greater depression symptoms. The original version of the scale (Radloff, 1991) has shown adequate internal consistency in the adult population (α = .84).

Finally, the number of new COVID‐19 cases and associated deaths by during the 7 days period prior to the assessment were obtained for each participant's country from https://covid19.who.int/.

### Procedure

The study was advertised on social media (Facebook and Twitter) on 26 March and 6 May 2020 as an investigation about the psychological effects of lockdown measures taken by governments. The anonymous assessment was conducted online and participants completed a consent form before participating. Participants were asked to complete the questionnaire only once and were encouraged to share the online link of the study with others. This could explain why we obtained participants from countries other than Ecuador, where the study was advertised. Data collection started on 26 March 2020 and was closed on 1 June 2020.

### Analyses

Since the sample included participants from several different countries with different cultural backgrounds and the 27‐item version of the CERQ has not been widely studied, its structure was analysed by performing a confirmatory factor analysis using maximum likelihood estimation. The internal consistency of the STAI, CES‐D and CERQ were also calculated for this sample. Following these analyses, several multiple regression models were tested using a stepwise (bidirectional) method selection. This method was chosen primarily due to the limited information available regarding the influence of specific variables on the presence of anxiety and depression during lockdown restrictions from COVID‐19. Finally, differences in the use and impact of CERS on anxiety and depression were investigated between male and female participants, and differences in the use of CERS between Groups 1 and 2. The latter analyses were performed to evaluate any difference between participants evaluated early versus later in the lockdown.

It is worth noting that the analyses related to sex and group differences were not pre‐registered.

## RESULTS

### Internal consistency of instruments

Internal consistency of all psychological instruments in the present study was calculated using Cronbach's α to facilitate a comparison with other studies and McDonald's *ω* which is a less biased alternative (Trizano‐Hermosilla & Alvarado, 2016). As observed in Table 2, all instruments presented acceptable to excellent reliability, with the lower scores deriving from all CERQ's sub‐scales, which contained three items each.

**TABLE 2:** Internal consistency of STAI, CES‐D and CERQ

	McDonald's *ω*	Cronbach's *α*
STAI	0.931	0.931
CES‐D	0.914	0.908
CERQ—other‐blame	0.848	0.842
CERQ—self‐blame	0.737	0.724
CERQ—acceptance	0.771	0.766
CERQ—rumination	0.763	0.763
CERQ—positive refocusing	0.817	0.816
CERQ—refocus on planning	0.834	0.833
CERQ—positive Reappraisal	0.866	0.862
CERQ—putting into perspective	0.793	0.792
CERQ—catastrophising	0.785	0.768

*Note*. CERQ = Cognitive emotion regulation questionnaire; CES‐D = center for epidemiological studies depression scale; STAI = state–trait anxiety inventory, state sub‐scale.

### Confirmatory factor analysis

The structure of the CERQ was analysed with the Spanish‐speaking sample used in the present study to evaluate if its original structure (the nine‐factor model) held in this multi‐cultural sample. A confirmatory factor analysis including the original nine‐factor structure was performed using maximum likelihood estimation. Although the model chi‐square was significant *χ* ^2^ (288) = 1302.77, *p* < .001, other indices, such as the root mean square error of approximation (RMSEA), the standardised root mean square residual (SRMR) and the comparative fit index (CFI), showed a good fit of the model: RMSEA = .073, CFI = .897, SRMR = .054. According to MacCallum et al. (1996) and Hu and Bentler (1999), the cut‐off for a good fit on these indices are RMSEA < .08, CFI ≥ .9 and SRMR < .08. Factor loadings for each item are high and significant, ranging from 0.62 to 1.02 (see Table 3). These results indicate that the CERQ is performing as expected in this sample measuring the nine cognitive emotion regulation strategies.

**TABLE 3:** Factor loadings from confirmatory factor analysis of the Cognitive Emotion Regulation Questionnaire (CERQ) in a Spanish‐speaking sample

				95% confidence interval	
Factor	Item	Estimate	*SE*	Lower	Upper
Self‐blame	1	0.633	0.039	0.557	0.708
	6	0.850	0.047	0.757	0.943
	20	0.819	0.046	0.729	0.910
Acceptance	2	0.888	0.043	0.804	0.971
	7	0.938	0.044	0.852	1.024
	21	0.855	0.047	0.762	0.947
Rumination	3	0.853	0.045	0.766	0.940
	8	0.871	0.047	0.779	0.963
	22	0.971	0.044	0.885	1.057
Positive refocusing	4	0.911	0.043	0.827	0.996
	9	0.992	0.044	0.906	1.078
	15	0.965	0.044	0.880	1.051
Other‐blame	5	0.770	0.037	0.698	0.842
	14	0.962	0.037	0.889	1.035
	27	0.836	0.036	0.765	0.907
Refocus on planning	10	0.954	0.039	0.877	1.032
	16	0.940	0.041	0.860	1.021
	23	0.949	0.040	0.870	1.028
Positive reappraisal	11	0.915	0.038	0.840	0.989
	17	1.015	0.039	0.938	1.092
	24	1.013	0.038	0.939	1.088
Putting into perspective	12	0.898	0.042	0.816	0.981
	18	0.958	0.042	0.875	1.040
	25	0.925	0.045	0.836	1.014
Catastrophising	13	0.976	0.039	0.900	1.053
	19	0.620	0.045	0.532	0.708
	26	0.973	0.039	0.897	1.049

### Descriptive statistics and correlations

Table 4 summarises descriptive statistics and correlations between the variables analysed. The first 45 participants completed an assessment protocol not including the CES‐D, due to an error in data collection.

**TABLE 4:** Descriptive statistics and Pearson's correlations between the analysed variables

	*N*	Mean	*SD*	1	2	3	4	5	6	7	8	9	10	11	12	13	14	15	16	17
1. CERQ—self‐blame	663	6.048	2.695	‐																
2. CERQ—acceptance	663	9.569	3.026	***0.321***	—															
3. CERQ—rumination	663	8.097	3.099	***0.489***	***0.531***	—														
4. CERQ—positive refocusing	663	8.997	3.177	***0.126***	***0.426***	***0.214***	—													
5. CERQ—refocus on planning	663	10.19	3.119	***0.271***	***0.590***	***0.430***	***0.565***	—												
6. CERQ—putting into perspective	663	10.214	3.141	***0.252***	***0.561***	***0.396***	***0.571***	***0.700***	—											
7. CERQ—catastrophising	663	6.504	2.893	***0.477***	***0.278***	***0.578***	***0.165***	***0.207***	***0.226***	—										
8. CERQ—other‐blame	663	5.848	2.789	***0.193***	***0.206***	***0.271***	***0.180***	0.076	***0.114***	***0.512***	—									
9. CERQ—positive reappraisal	663	10.615	3.158	***0.195***	***0.546***	***0.332***	***0.622***	***0.799***	***0.781***	**0.094**	0.041	—								
10. Depression	618	19.605	11.509	***0.354***	**0.080**	***0.378***	−0.061	−0.034	−0.044	***0.508***	***0.315***	***−0.176***	—							
11. Anxiety	663	26.374	11.803	***0.193***	0.005	***0.283***	**−0.090**	**−0.078**	−0.055	***0.379***	***0.220***	***−0.194***	***0.592***	—						
12. Information seeking	662	2.819	3.969	0.040	−0.044	0.047	−0.022	−0.006	−0.024	−0.009	0.019	−0.005	0.029	**0.083**	—					
13. People in the house	663	4.179	1.938	0.042	0.050	**0.082**	0.033	0.063	0.006	**0.093**	**0.091**	0.015	0.073	0.041	0.014	—				
14. Risk perception	663	2.789	1.147	***0.132***	0.038	**0.079**	0.074	**0.104**	0.061	***0.154***	0.066	0.043	**0.094**	***0.193***	**0.099**	0.009	—			
15. Age	663	30.03	11.302	**−0.087**	***−0.154***	***−0.275***	0.068	−0.006	−0.022	***−0.195***	**−0.092**	0.054	***−0.240***	***−0.147***	0.068	*−**0.132***	0.048	—		
16. Number of hobbies	663	5.676	1.92	0	0.076	**0.091**	0.022	**0.108**	0.058	0.056	0	**0.097**	−0.010	−**0.095**	−0.024	−0.013	−0.047	*−**0.181***		
17. Mean number of new cases	663	449.14	1348.29	−0.049	−0.074	−0.038	−0.019	−0.051	−0.028	−0.005	−0.017	−0.053	−0.015	0.005	−0.019	−0.058	0.020	−0.010	0.058	
18. Mean number of deaths	663	63.22	109.56	−0.050	***−0.122***	**−0.091**	−0.032	***−0.114***	−0.072	−0.010	−0.061	**−0.107**	−0.045	−0.007	**−0.094**	−0.052	0.018	−0.004	0.004	***0.878***

*Note:* Significant correlations (*p* < .05) are presented in bold. Correlations presented in bold and italics are significant after correcting for multiple comparisons using the Bonferroni procedure (0.05/16 = 0.003125).

### Multiple linear regression models

**Figure 1:**
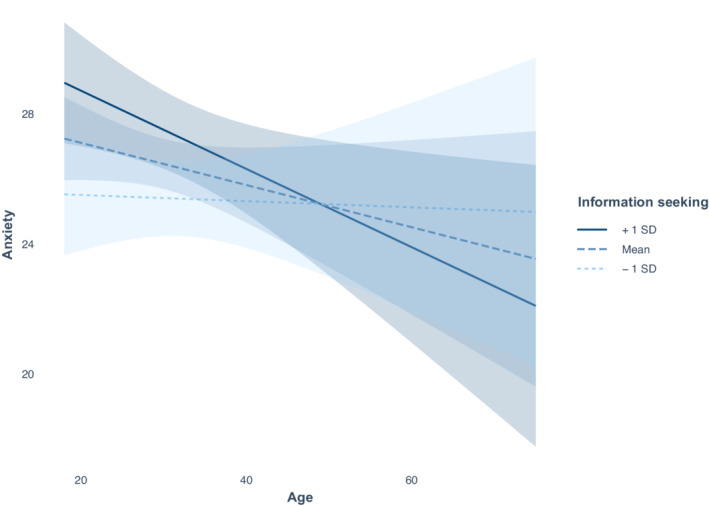
Slopes for age predicting anxiety depending on information seeking.

Two stepwise model selection analyses were performed to identify variables predicting the appearance of anxiety and depression symptoms. In both models, all nine CERS were included, as well as group (group 1 served as reference), perceived risk of getting infected with the virus (risk perception), age, sex (males served as reference) if they had a significant other, number of persons living in the same house during lockdown restrictions, number of hobbies, number of times they looked for information about COVID‐19 during the day (information seeking), the mean number of new cases and deaths due to COVID‐19 in the 7 days prior to their assessment, and the interaction between age and information seeking. This interaction was included since older adults might have a more limited access to new technology to seek for updated information (e.g., Facebook, WhatsApp, YouTube) and, therefore, be less affected by COVID‐19‐related news. The resulting models explained 29% of variance in anxiety symptoms (*F*[11, 637] = 23.72, *p* < .001), and 39% of variance in depression symptoms (*F*[8, 596] = 48.25, *p* < .001). Table 5 presents the results of each model. As depicted in Figure 1, higher information seeking was associated with higher anxiety in younger adults but not in older adults.

**TABLE 5:** Multiple regression models for anxiety and depression symptoms

Model and predictors	B	95% Confidence Interval	SE	*β*	t	p
Anxiety						
Intercept	22.536	[16.93, 28.141]	2.855		7.894	<.001
Rumination	.704	[0.366, 1.042]	0.172	.186	4.090	<.001
Catastrophising	1.109	[0.772, 1.447]	0.172	.273	6.457	<.001
Positive Reappraisal	−1.071	[−1.337, −0.804]	0.136	−.289	−7.895	<.001
Risk perception	1.536	[0.852, 2.221]	0.349	.150	4.405	<.001
Number of hobbies	−.656	[−1.068, −0.244]	0.210	−.108	−3.126	.002
Information seeking	.680	[0.111, 1.25]	0.290	.231	2.345	.019
Having a significant other	1.747	[−0.271, 3.764]	1.027	.067	1.700	.090
Age	−.026	[−0.123, 0.072]	0.050	−.025	−0.519	.604
Group	−1.234	[−2.845, 0.376]	0.820	−.053	−1.505	.133
Sex	3.029	[1.372, 4.686]	0.844	.124	3.590	<.001
Age * information seeking	−.014	[−0.028, 0]	0.007	−.004	−1.914	.056
Depression						
Intercept	12.801	[8.503, 17.1]	2.189		5.849	<.001
Self‐blame	.610	[0.28, 0.94]	0.168	.142	3.630	<.001
Rumination	.667	[0.34, 0.994]	0.167	.180	4.002	<.001
Catastrophising	1.173	[0.811, 1.536]	0.185	.295	6.356	<.001
Other‐blame	.389	[0.087, 0.691]	0.154	.095	2.528	.012
Positive reappraisal	−1.152	[−1.397, −0.906]	0.125	−.320	−9.204	<.001
Risk perception	.588	[−0.05, 1.227]	0.325	.059	1.811	.071
Age	−.106	[−0.18, −0.032]	0.037	−.097	−2.826	.005
Sex	1.977	[0.418, 3.537]	0.794	.082	2.490	.013

*Note*: *B* = unstandardised beta coefficient; *β* = standardised beta coefficient.

#### Sex differences in cognitive emotion regulation, anxiety and depression

Differences between male and female participants in the use of CERS were analysed using Welch's *t* tests. Significant differences were found in the use of self‐blame (*t*[478.96] = 2.729, *p* = .007) and Rumination (*t*[485.17] = −2.494, *p* = .013), with males (*M* = 6.42, *SD* = 2.75) scoring higher in self‐blame than females (*M* = 5.82, *SD* = 2.64), and females (*M* = 8.31, *SD* = 3.06) scoring higher on Rumination than males (*M* = 7.68, *SD* = 3.14). Sex differences were also analysed for symptoms of depression and anxiety. Female participants scored significantly higher than male participants in both depression (mean males [*SD*] = 18.1 [11.51], mean females [*SD*] = 20.42 [11.5]) and anxiety (mean males [*SD*] = 23.95 [11.73]; mean females [*SD*] = 27.74 [11.6]). However, when controlling for multiple comparisons using the Bonferroni procedure, only differences in anxiety achieved significance. Results from this analysis can be found in Table S1 in Supporting Information.

**Figure 2:**
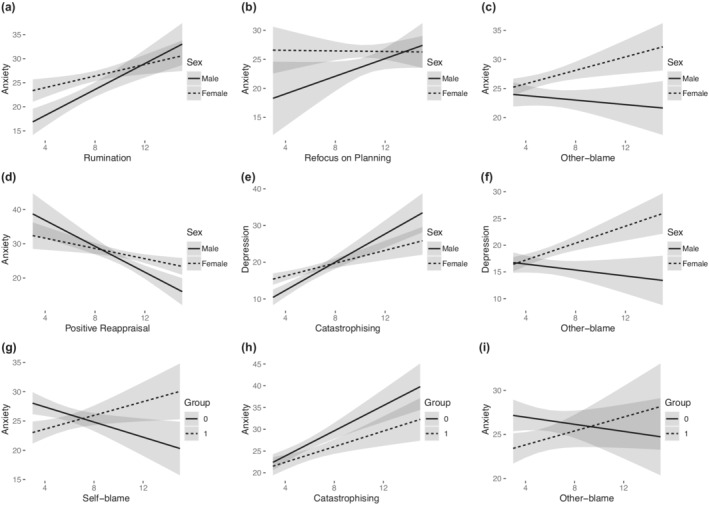
Slopes depicting interaction effects from cognitive emotion regulation strategies (CERS) with group and sex on the prediction of anxiety and depression. (a) Slopes for Rumination depending on sex, (b) slopes for refocus on planning depending on sex, (c) slopes for other‐blame depending on sex, (d) slopes for positive reappraisal depending on sex, (e) slopes for catastrophising depending on sex, (f) slopes for other‐blame depending on sex, (g) slopes for self‐blame depending on group, (h) slopes for catastrophising depending on group, (i) slopes for other‐blame depending on group.

Sex differences in the moderating role of CERS in anxiety and depression were also analysed in two stepwise regression models, where the interactions between CERS and sex (males as reference) were analysed in addition to their main effects. For anxiety, the resulting model predicted 27% of variance (*F*[10, 645] = 23.31, *p* < .001) and was significantly better than a model including only CERS (*F*[8, 637] = 3.954, *p* < .001). The resulting model is presented in Table S2 and includes the interaction between sex and: rumination (*B* = −.744, Figure 2a), refocus on planning (*B* = −.786, Figure 2b), other‐blame (.771, Figure 2c), and positive reappraisal (*B* = 1.142, Figure 2d). The model also included Rumination (*B* = 1.347), catastrophising (*B* = 1.008) and positive reappraisal (*B* = −1.89) as significant predictors (intercept *B* = 23.519).

For depression, the final model predicted 40% of its variance (*F*[8, 596] = 49.25, *p* < .001) and was significantly better than a model including only CERS (*F*[3, 596] = 7.796, *p* < .001). The resulting model is presented in Table S2 and includes the interaction between sex and: catastrophising (*B* = −1.049, Figure 2), and other‐blame (*B* = 1.07, Figure 2f). The model also included self‐blame (*B* = .616), rumination (*B* = .76), catastrophising (*B* = 1.918) and positive reappraisal (*B* = −1.18) as significant predictors (intercept *B* = 9.905).

As observed from the interactions, male and female participants could respond very differently to a number of CERS.

#### The use of CERS across time

A set of Welsch's *t* tests between the first and second group of responders was performed to determine differences in the use of CERS. After adjusting for multiple comparisons using the Bonferroni procedure, significant differences were found in the use of Refocus on Planning (*t* = 4.032, *df* = 630.561, *p* < .001, mean group 1 [*SD*] = 10.64 [2.95], mean group 2 (*SD*) = 9.66 [3.24]), putting into perspective (*t* = 3.336, *df* = 636.121, *p* < .001, mean group 1 [*SD*] = 10.59 [3], mean group 2 [*SD*] = 9.78 [3.22]), positive reappraisal (*t* = 3.849, *df* = 631.62, *p* < .001, mean group 1 [*SD*] = 11.05 [2.99], mean group 2 [*SD*] = 10.11 [3.28]) and acceptance (*t* = 2.8, *df* = 649.46, *p* = .005, mean group 1 [*SD*] = 9.87 [3.01], mean group 2 [*SD*] = 9.21 [3]).

The mediating effect of group on the impact of CERS on anxiety and depression was analysed by including the interaction between CERS and group (group 1 as reference) in stepwise regression models. In the case of anxiety, the final model predicted 25% if its variance (*F*[10, 638] = 21.52, *p* < .001) and was significantly better than a model including only CERS (*F*[7, 638] = 2.854, *p* = .006). The resulting model is presented in Table S2 and includes the interaction between group and: Self‐blame (*B* = 1.229, Figure 2g), catastrophising (*B* = −.552, Figure 2h) and other‐blame (*B* = .596, Figure 2i). The model also included Rumination (*B* = .862), catastrophising (*B* = 1.449), positive reappraisal (*B* = −1.028) and group (*B* = −8.354) as significant predictors (intercept *B* = 28.966). In the case of depression, the resulting model was not better than a model including only CERS (*F*[7, 592] = 1.13, *p* = .346).

## DISCUSSION

Before analysing the predictive value of our independent variables in anxiety and depression symptoms, we analysed the psychometric properties of the CERQ, CES‐D and STAI. This was particularly important since our sample included participants of different cultural backgrounds and the 27‐item version of the CERQ has not been widely‐studied. It is worth noting that, in a study investigating the psychometric properties of the CERQ in a Spanish sample (Domínguez‐Sánchez et al., 2013), the authors found two of the items to be potentially affected by culture. Our results indicated that the CERQ maintained its 9‐factor structure in our sample, supporting the use of this instrument across cultures. Although we have not directly compared the performance of the 27‐item versus the 36‐item version of the CERQ, the elimination of certain items appears to improve the performance of the instrument, since we have not found any issues related to poor performance of items as other studies using Spanish speaking samples have (e.g., Dominguez & Medrano, 2016; Domínguez‐Sánchez et al., 2013). The internal consistencies of the CERQ, STAI and CES‐D were found to be acceptable to excellent, similar to studies with other samples (Barnes et al., 2002; Domínguez‐Sánchez et al., 2013; Radloff, 1991).

After confirming the psychometric properties of the instruments, we analysed the predictive value of several variables related to the lockdown and CERS on psychological distress, more specifically, on anxiety and depression symptoms. From the resulting models, it is clear that CERS play a predominant role in predicting both anxiety and depression. In fact, models exclusively composed of CERS predict 23% of the variance of anxiety, and 37% in the case of depression, meaning that the inclusion of the other behavioural and demographic variables only improves the models' predictive value by 6 and 2%, respectively.

Among the emotion regulation strategies investigated, positive reappraisal, catastrophising and rumination appeared as significant predictors for both the anxiety and depression models, with positive reappraisal serving as a protective factor and the other two tending to aggravate negative symptoms. Prior studies (e.g., Garnefski & Kraaij, 2006) have already reported the relationship of rumination, catastrophising and positive reappraisal with anxiety and depression, and this relationship seems to be stable across populations (Garnefski & Kraaij, 2006; Potthoff et al., 2016).

The outbreak of COVID‐19 provided fertile ground for catastrophising and ruminating, particularly when lockdown measures were being undertaken by governments and the news and social media were plagued with information eliciting negative emotions (Aslam et al., 2020). It is possible that this situation increased the importance of CERS in determining psychological distress, particularly when compared to the other predictors included in the model. Despite the abundance of information related to COVID‐19, it is noteworthy that the group evaluated by the end of the assessment phase (group 2) was less anxious and that the effect of CERS was different between time of assessment, with self‐blame and other‐blame having a negative impact on the second group and catastrophising a higher impact on the first group. It is possible that, despite the increase in deaths and infections, the population was less scared of the virus. This is supported by the decreased effect of catastrophising by the second round of data collection. Nevertheless, it is important to note that this study was cross‐sectional in nature, and any conclusions about the effects of the passage of time are speculative. Future studies may wish to include longitudinal assessments of changes in coping strategies during periods of national crisis.

Sex also appeared as a significant mediator of the impact of CERS on psychological distress, with strategies such as rumination, catastrophising or positive reappraisal having a higher impact over psychological distress on male participants, and other‐blame (increasing both anxiety and depression) on female participants. Previous studies (Wu et al., 2016) have observed neural differences on the effects of emotion regulation between male and female participants; however, it is possible that idiosyncratic variables might be playing an important role in this case, since the studied strategies involved a cognitive component.

According to our results, emotion regulation played a key role in the experience of anxiety and depression symptoms during the first few months of the pandemic when lockdown measures were taken and public response was marked with negative emotions. An examination of the descriptive analysis reveals that the mean score of depression obtained from the CES‐D in the sample is above the recommended cut‐off point of 16, which would indicate “cases of depression” according to Weissman et al. (1977, p. 206). In fact, 50% of the sample obtained a score of 17 or higher in this scale. This score reveals to some extent the emotional impact that the lockdown and the pandemic had in the population during the first months, although it is important to note that only self‐report measures were obtained, and no clinical diagnoses are available. In addition to CERS, other variables were analysed as possible predictors of anxiety and depression during the lockdown, and, in line with other studies (i.e., Fullana et al., 2020; Gao et al., 2020; Rubaltelli et al., 2020), the number of hobbies, information seeking and age were found to be significant predictors during lockdown. However, it is important to note that their contribution to the models was not as strong as those presented by the CERS. These results highlight the impact that CERS had on developing anxiety and depression symptoms, suggesting that psychological interventions should focus significant efforts on promoting the use of positive reappraisal and reducing the use of catastrophising and rumination, among the strongest predictors in both models. Even more, these findings can serve as a pivot for developing psychological interventions focused on coping with highly stressful events in general and lockdown scenarios in particular.

## Supporting information


**Table S1.** Differences between male and female participants in cognitive emotion regulation strategies, anxiety and depression
**Table S2.** Multiple regression models predicting anxiety and depression with CERS and their interaction with group or sex as preditors

## References

[ijop12818-bib-0001] Aslam, F. , Awan, T. M. , Syed, J. H. , Kashif, A. , & Parveen, M. (2020). Sentiments and emotions evoked by news headlines of coronavirus disease (COVID‐19) outbreak. Humanities and Social Sciences Communications, 7(1), 23. 10.1057/s41599-020-0523-3

[ijop12818-bib-0002] Baqutayan, S. (2011). Stress and social support. Indian Journal of Psychological Medicine, 33(1), 29–34. 10.4103/0253-7176.85392 22021950 PMC3195151

[ijop12818-bib-0003] Barnes, L. L. B. , Harp, D. , & Jung, W. S. (2002). Reliability generalization of scores on the Spielberger state‐trait anxiety inventory. Educational and Psychological Measurement, 62(4), 603–618. 10.1177/0013164402062004005

[ijop12818-bib-0031] Buela‐Casal, G. , Guillén‐Riquelme, A. & Seisdedos Cubero, N. (2015). *STAI, Cuestionario de Anisedad Estado‐Rasgo Manual* (9th ed.), TEA Ediciones.

[ijop12818-bib-0004] Çivitci, A. (2015). The moderating role of positive and negative affect on the relationship between perceived social support and stress in college students. Educational Sciences: Theory & Practice, 15(3), 565–573. 10.12738/estp.2015.3.2553

[ijop12818-bib-0005] Dominguez, S. , & Medrano, L. A. (2016). Invarianza factorial del Cognitive Emotional Regulation Questionarie (CERQ) en universitarios limeños y cordobeses. Universitas Psychologica, 15(1), 15–24. 10.11144/Javeriana.upsy15-1.ifce

[ijop12818-bib-0006] Domínguez‐Sánchez, F. J. , Lasa‐Aristu, A. , Amor, P. J. , & Holgado‐Tello, F. P. (2013). Psychometric properties of the Spanish version of the cognitive emotion regulation questionnaire. Assessment, 20(2), 253–261. 10.1177/1073191110397274 21467092

[ijop12818-bib-0007] Faul, F. , Erdfelder, E. , Buchner, A. , & Lang, A.‐G. (2009). Statistical power analyses using G*power 3.1: Tests for correlation and regression analyses. Behavior Research Methods, 41, 1149–1160. 19897823 10.3758/BRM.41.4.1149

[ijop12818-bib-0008] Fullana, M. A. , Hidalgo‐Mazzei, D. , Vieta, E. , & Radua, J. (2020). Coping behaviors associated with decreased anxiety and depressive symptoms during the COVID‐19 pandemic and lockdown. Journal of Affective Disorders, 275, 80–81. 10.1016/j.jad.2020.06.027 32658829 PMC7329680

[ijop12818-bib-0009] Gao, J. , Zheng, P. , Jia, Y. , Chen, H. , Mao, Y. , Chen, S. , Wang, Y. , Fu, H. , & Dai, J. (2020). Mental health problems and social media exposure during COVID‐19 outbreak. PLoS One, 15(4), e0231924. 10.1371/journal.pone.0231924 32298385 PMC7162477

[ijop12818-bib-0010] Garnefski, N. , & Kraaij, V. (2006). Relationships between cognitive emotion regulation strategies and depressive symptoms: A comparative study of five specific samples. Personality and Individual Differences, 40(8), 1659–1669. 10.1016/j.paid.2005.12.009

[ijop12818-bib-0011] Garnefski, N. , & Kraaij, V. (2007). The cognitive emotion regulation questionnaire. European Journal of Psychological Assessment, 23(3), 141–149. 10.1027/1015-5759.23.3.141

[ijop12818-bib-0012] Garnefski, N. , & Kraaij, V. (2018). Specificity of relations between adolescents' cognitive emotion regulation strategies and symptoms of depression and anxiety. Cognition and Emotion, 32(7), 1401–1408. 10.1080/02699931.2016.1232698 27648495

[ijop12818-bib-0013] González‐Forteza, C. , Jiménez‐Tapia, J. A. , Lamos‐Lira, L. , & Wagner, F. A. (2008). Application of the revised version of the Center of Epidemiological Studies Depression Scale in adolescent students from Mexico City. Salud Publica de Mexico, 50, 292–299. 10.1590/s0036-36342008000400007 18670720 PMC13472125

[ijop12818-bib-0014] Holgado‐Tello, F. P. , Amor, P. J. , Lasa‐Aristu, A. , Domínguez‐Sánchez, F. J. , & Delgado, B. (2018). Two new brief versions of the cognitive emotion regulation questionnaire and its relationships with depression and anxiety. Anales de Psicología, 34(3), 458–464. 10.6018/analesps.34.3.306531

[ijop12818-bib-0015] Hu, L. , & Bentler, P. M. (1999). Cutoff criteria for fit indexes in covariance structure analysis: Conventional criteria versus new alternatives. Structural Equation Modeling: A Multidisciplinary Journal, 6(1), 1–55. 10.1080/10705519909540118

[ijop12818-bib-0016] Hu, T. , Zhang, D. , Wang, J. , Mistry, R. , Ran, G. , & Wang, X. (2014). Relation between emotion regulation and mental health: A meta‐analysis review. Psychological Reports, 114(2), 341–362. 10.2466/03.20.PR0.114k22w4 24897894

[ijop12818-bib-0018] Kraiss, J. T. , ten Klooster, P. M. , Moskowitz, J. T. , & Bohlmeijer, E. T. (2020). The relationship between emotion regulation and well‐being in patients with mental disorders: A meta‐analysis. Comprehensive Psychiatry, 102, 152189. 10.1016/j.comppsych.2020.152189 32629064

[ijop12818-bib-0019] MacCallum, R. C. , Browne, M. W. , & Sugawara, H. M. (1996). Power analysis and determination of sample size for covariance structure modeling. Psychological Methods, 1(2), 130–149.

[ijop12818-bib-0020] Picó‐Pérez, M. , Radua, J. , Steward, T. , Menchón, J. M. , & Soriano‐Mas, C. (2017). Emotion regulation in mood and anxiety disorders: A meta‐analysis of fMRI cognitive reappraisal studies. Progress in Neuro‐Psychopharmacology and Biological Psychiatry, 79, 96–104. 10.1016/j.pnpbp.2017.06.001 28579400

[ijop12818-bib-0021] Potthoff, S. , Garnefski, N. , Miklósi, M. , Ubbiali, A. , Domínguez‐Sánchez, F. J. , Martins, E. C. , Witthöft, M. , & Kraaij, V. (2016). Cognitive emotion regulation and psychopathology across cultures: A comparison between six European countries. Personality and Individual Differences, 98, 218–224. 10.1016/j.paid.2016.04.022

[ijop12818-bib-0022] Radloff, L. S. (1991). The use of the Center for Epidemiologic Studies Depression Scale in adolescents and young adults. Journal of Youth and Adolescence, 20(2), 149–166. 10.1007/BF01537606 24265004

[ijop12818-bib-0023] Rodas, J. A. , Jara‐Rizzo, M. , & Oleas, D. (2021). Emotion regulation, psychological distress and demographic characteristics from an Ecuadorian sample: Data from the lockdown due to COVID‐19. Data in Brief, 37, 107182. 10.1016/j.dib.2021.107182 34136605 PMC8181774

[ijop12818-bib-0024] Rubaltelli, E. , Tedaldi, E. , Orabona, N. , & Scrimin, S. (2020). Environmental and psychological variables influencing reactions to the COVID‐19 outbreak. British Journal of Health Psychology, 25(4), 1020–1038. 10.1111/bjhp.12473 32951244 PMC7537169

[ijop12818-bib-0025] Spielberger, C. D. (2010). State‐trait anxiety inventory. In I. B. Weiner & W. E. Craighead (Eds.), The Corsini encyclopedia of psychology (p. corpsy0943. John Wiley & Sons, Inc.. 10.1002/9780470479216.corpsy0943

[ijop12818-bib-0026] Trizano‐Hermosilla, I. , & Alvarado, J. M. (2016). Best alternatives to Cronbach's alpha reliability in realistic conditions: Congeneric and asymmetrical measurements. Frontiers in Psychology, 7, 769. 10.3389/fpsyg.2016.00769 27303333 PMC4880791

[ijop12818-bib-0027] Weissman, M. M. , Sholomskas, D. , Pottenger, M. , Prusoff, B. A. , & Locke, B. Z. (1977). Assessing depressive symptoms in five psychiatric populations: A validation study. American Journal of Epidemiology, 106(3), 203–214. 10.1093/oxfordjournals.aje.a112455 900119

[ijop12818-bib-0028] Wittig, R. M. , Crockford, C. , Weltring, A. , Langergraber, K. E. , Deschner, T. , & Zuberbühler, K. (2016). Social support reduces stress hormone levels in wild chimpanzees across stressful events and everyday affiliations. Nature Communications, 7(1), 13361. 10.1038/ncomms13361 PMC5097121 27802260

[ijop12818-bib-0029] Wu, Y. , Li, H. , Zhou, Y. , Yu, J. , Zhang, Y. , Song, M. , Qin, W. , Yu, C. , & Jiang, T. (2016). Sex‐specific neural circuits of emotion regulation in the centromedial amygdala. Scientific Reports, 6(1), 23112. 10.1038/srep23112 27004933 PMC4804331

[ijop12818-bib-0030] Zajenkowski, M. , Jonason, P. K. , Leniarska, M. , & Kozakiewicz, Z. (2020). Who complies with the restrictions to reduce the spread of COVID‐19?: Personality and perceptions of the COVID‐19 situation. Personality and Individual Differences, 166, 110199. 10.1016/j.paid.2020.110199 32565591 PMC7296320

