# Supplementary material for: Cognitive emotion regulation strategies and psychological distress during lockdown due to COVID‐19
Source: Int J Psychol. 2021 Nov 7;57(3):315–24. doi: 10.1002/ijop.12818 (PMC8652999; doi:10.1002/ijop.12818)
Supplement: Supplementary file 1 — Table S1. Differences between male and female participants in cognitive emotion regulation strategies, anxiety and depression Table S2. Multiple regression models predicting anxiety and depression with CERS and their interaction with group or sex as preditors [file IJOP-57-315-s001.pdf]

Supplemental Materials

Table S1

Differences Between Male and Female Participants in Cognitive Emotion Regulation Strategies, Anxiety and Depression

	Male		Female				
	**Mean**	**SD**	**Mean**	**SD**	**t**	**p**	**g**
Self-blame	6.423	2.75	5.823	2.638	2.729	0.007	0.222
Acceptance	9.481	3.166	9.604	2.956	-0.491	0.624	-0.04
Rumination	7.682	3.142	8.312	3.061	-2.494	0.013	-0.203
Positive Refocusing	8.866	3.066	9.07	3.261	-0.799	0.425	-0.064
Refocus on Planning	10.272	3.247	10.129	3.058	0.552	0.581	0.045
Putting into Perspective	10.067	3.124	10.302	3.152	-0.925	0.355	-0.075
Catastrophising	6.364	2.86	6.59	2.913	-0.967	0.334	-0.078
Other-blame	5.929	2.928	5.796	2.714	0.574	0.566	0.047
Positive Reappraisal	10.669	3.202	10.564	3.141	0.41	0.682	0.033
Anxiety	23.95	11.729	27.736	11.595	-3.996	<.001	-0.324
Depression	18.102	11.508	20.423	11.496	-2.383	0.018	-0.202

Note: Welch's *t* tests were used. Following the Bonferroni procedure for multiple comparisons, statistical significance is achieved with $p \leq 0.0045$.

Table S2

Multiple Regression Models Predicting Anxiety and Depression with CERS and their Interaction with Group or Sex as preditors

Model and predictors	B	95% Confidence Interval	Standard Error	β	t	p
**Resulting model predicting anxiety with CERS and their interaction with sex as predictors**						
Intercept	23.519	0.000	2.640		8.909	0.000
Acceptance	-0.286	0.000	0.182	-0.074	-1.574	0.116
Rumination	1.347	0.000	0.272	0.356	4.951	0.000
Refocus on Planning	0.759	0.000	0.412	0.202	1.841	0.066
Catastrophising	1.008	0.000	0.193	0.248	5.226	0.000
Other-blame	-0.194	0.000	0.252	-0.046	-0.772	0.441
Positive Reappraisal	-1.890	0.000	0.405	-0.510	-4.673	0.000
Sex	0.197	0.000	3.394	0.008	0.058	0.954
Rumination * Sex	-0.744	0.000	0.311	-0.192	-2.394	0.017
Refocus on Planning * Sex	-0.786	0.000	0.489	-0.207	-1.608	0.108

Other-blame * Sex	0.771	0.000	0.310	0.205	2.489	0.013
Positive Reappraisal * Sex	1.142	0.000	0.473	0.280	2.416	0.016
**Resulting model predicting depression with CERS and their interaction with sex as predictors**						
Intercept	9.905	0.000	1.969		5.031	0.000
Self-blame	0.616	0.000	0.167	0.206	3.691	0.000
Rumination	0.760	0.000	0.162	0.206	4.683	0.000
Catastrophising	1.918	0.000	0.287	0.482	6.691	0.000
Other-blame	-0.278	0.000	0.247	-0.068	-1.129	0.259
Positive Reappraisal	-1.180	0.000	0.124	-0.328	-9.542	0.000
Sex	2.767	0.000	2.079	0.115	1.331	0.184
Catastrophising * Sex	-1.049	0.000	0.317	-0.245	-3.307	0.000
Other-blame * Sex	1.070	0.000	0.315	0.289	3.398	0.000
**Resulting model predicting anxiety with CERS and their interaction with group as predictors**						
(Intercept)	28.966	0.000	2.268		12.769	0.000
Self-blame	-0.644	0.000	0.250	-0.147	-2.573	0.010
Acceptance	-0.252	0.000	0.179	-0.065	-1.406	0.160
Rumination	0.862	0.000	0.188	0.227	4.573	0.000
Catastrophising	1.449	0.000	0.279	0.356	5.189	0.000
Other-blame	-0.201	0.000	0.235	-0.048	-0.855	0.393
Positive Reappraisal	-1.028	0.000	0.155	-0.277	-6.636	0.000
Group	-8.354	0.000	2.488	-0.356	-3.358	0.000
Self-blame * Group	1.229	0.000	0.349	0.280	3.526	0.000
Catastrophising * Group	-0.552	0.000	0.368	-0.142	-1.501	0.134
Other-blame * Group	0.596	0.000	0.341	0.157	1.748	0.081

Note: B = unstandardised beta coefficient; β = standardised beta coefficient.
